# Adherence to veterinary recommendations for ectoparasiticides purchased by cat owners in the USA

**DOI:** 10.1186/s13071-020-04415-5

**Published:** 2020-10-31

**Authors:** Robert Lavan, Rob Armstrong, Dorothy Normile, Wendy Vaala

**Affiliations:** 1 Center for Observational and Real-World Evidence, Merck & Co., Inc., Kenilworth, NJ USA; 2 Merck Animal Health, 2 Giralda Farms, Madison, NJ USA

**Keywords:** Adherence, Bravecto, Cat, Ectoparasiticide, Flea, Isoxazolines, Tick

## Abstract

**Background:**

Safe and effective flea and tick treatment options for cats are important in companion animal practice because of feline ectoparasite infestation prevalence and the potential for parasitic disease transmission. Retrospective cat owner purchasing transactions at United States of America (USA) veterinary clinics were obtained for three topical feline flea and tick ectoparasiticides. One medication, fluralaner, had a 12-week redosing interval, while two other medications (fipronil/s-methoprene/pyriproxyfen; imidacloprid/pyriproxyfen) were approved for monthly redosing. The annual number of doses purchased by cat owners was determined for each of the three medications and then compared between medications. The objective was to evaluate whether 12-week retreatment intervals resulted in a different duration of coverage compared to monthly treatments for ectoparasiticide products.

**Methods:**

Study results were obtained by analyzing the transactional records from a commercial database derived from veterinary practice management software. The study database consisted of cat owner purchasing records from January 2017 through June 2019 from 671 veterinary practices representing 41,630 cats.

**Results:**

Cat owners purchased an average of 1.5 doses of fluralaner per year which, based on a 12-week redosing interval, provides 4.2 months of treatment coverage. Cat owners who used monthly flea and tick medications respectively purchased 3.6 months (fipronil/s-methoprene/pyriproxyfen combination) and 2.8 months (imidacloprid/pyriproxyfen) annually of each of the two medications. Average yearly cat owner purchases of fluralaner provide a significantly longer duration of coverage than for cat owners purchasing fipronil/s-methoprene/pyriproxyfen (17% more) or imidacloprid/pyriproxyfen (50% more).

**Conclusions:**

Cat owners who obtained a flea and tick treatment with a 12-week redosing interval (fluralaner) protected their cats for up to 17% or 50% longer duration each year, respectively, compared to the duration of protection obtained by cat owners who used a medication re-dosed monthly. Cat owners should increase their duration of flea and tick coverage to come closer to achieving veterinary recommendations.
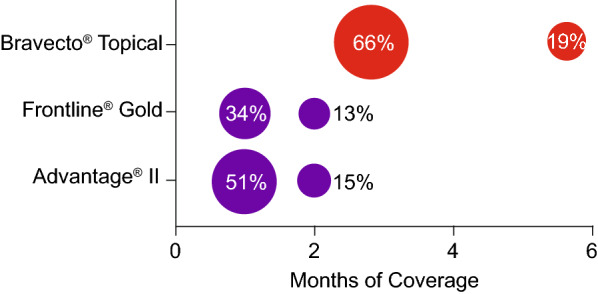

## Background

In primary care veterinary practice, flea and tick control is an essential component of the preventive healthcare strategy for cats. This is because of the high prevalence of flea infestation, the year-round risk of ticks, the risk of flea allergy dermatitis (FAD), the pathogens that fleas and ticks vector, and the introduction of fleas into the home [1, 2].

Fleas, particularly *Ctenocephalides felis felis*, are very common and perhaps the most clinically important ectoparasite of cats and dogs worldwide [3–5]. Flea-related diseases could account for more than half of dermatologic cases presented to companion animal veterinarians [5–7]. Results of European studies confirm that levels of feline flea infestation are variable, and can exceed 70% in an untreated population [6]. A study of free-roaming cats in the central USA in 2014 found fleas on 71.6% and ticks on 18.7% of trapped cats [8]. A multi-center study conducted at veterinary facilities in nine European countries evaluated 1519 client-owned cats untreated with ectoparasiticides in the preceding month [9] and found an overall 15.5% flea infestation prevalence. A flea infestation prevalence of 22.9% was reported in feline patients at 13 veterinary practices in Hungary [10], and a 21.1% prevalence at 31 practices in the UK [11]. In both the Hungarian and UK studies, the flea infestation prevalence in cats was considerably greater than the prevalence in dogs evaluated concurrently at the same sites. Moreover, in the UK study the prevalence of FAD lesions in cats (8.0%) was significantly greater (*P* < 0.001) than in dogs (6.8%). Approximately half of the pet owners in the UK study whose pets harbored fleas were unaware that their animals were infested. Other investigators confirm that apparently healthy cats can harbor low levels of flea infestation, in some cases the lack of flea awareness is apparently a result of overgrooming by the cat [12]. Feral cats, untreated rural cats and indoor-outdoor cats may have considerably higher levels of infestation, associated with their increased outdoor exposure to fleas, than indoor cats [3, 10].

Fleas and ticks also carry and transmit an assortment of vector-borne pathogens that can infect cats and people, including *Bartonella* spp., *Ehrlichia* spp., *Rickettsia* spp., *Mycoplasma* spp., as well as cestode, protozoan, and filarial endoparasites [1, 2, 4, 13, 14]. Investigators have found that as many as 80% of fleas collected from cat populations harbor feline or human pathogens of importance [15].

Safe and effective feline flea and tick treatment options are therefore an important component of the companion animal armamentarium. The isoxazoline class of parasiticide compounds were introduced in 2013 for use in dogs and 2 years later for use in cats. Fluralaner (Bravecto^®^, Merck & Co., Inc., Madison, NJ, USA) is a long-acting isoxazoline class molecule available in oral and topical formulations for use in dogs and in a topical formulation for use on cats, and is indicated for 12 week control of fleas and up to 12 weeks against a variety of tick species [16–21]. A recent field study found that a single topical dose of fluralaner reduced flea populations on household cats by 96.6% within 7 days and by 100% at 12 weeks after treatment [13, 22].

Fluralaner kills newly emerging adult fleas on the host before they start laying eggs, which in untreated animals would fall off the host into the environment and develop into flea larvae and pupae. Interrupting on-animal egg production disrupts the flea life-cycle, depleting the flea population in the home surroundings and preventing subsequent re-infestation of the host [23]. Fluralaner has demonstrated onset of flea adulticidal activity within 2 hours after topical administration [16]. As home environment studies have indicated [13, 24], a rapid speed of kill and long duration of action prevent re-infestation of the host and home environment by eliminating all flea life stages from the environment.

In an effort to sample the veterinarian recommendations for flea and tick prevention in dogs, approximately 30 veterinarians in each of the USA, UK and Australia were surveyed as part of a larger pet owner survey [25]. For dogs, veterinarians recommended approximately 12 months of protection against fleas and 9–12 months protection against ticks per year. The Companion Animal Parasite Council recommends year-round ectoparasite control for dogs and cats [26].

Feline flea and tick control before the approval of fluralaner commonly involved ectoparasiticide administration to cats at monthly intervals. Weak owner adherence with recommended flea treatments may lead to a failure or delay in administering follow-on doses at the required time. This lack of adherence leads to persistence of the flea population in the household, and failure to follow recommended treatment recommendations is identified as the most common cause of flea treatment failure [27]. Lack of treatment adherence is often followed by a perceived lack of efficacy and is a vital issue in clinical practice because it affects the owners’ confidence in the prescriber and medication as well as the actual treatment success.

One option for veterinary practitioners to potentially increase adherence to veterinary recommendations for flea and tick protection is through prescription of an extended duration product. This approach requires the cat owner to administer fewer doses over the same period of time. Prior studies across an array of drug classes in human and veterinary medicine have consistently shown an inverse relationship between treatment adherence and the complexity (particularly higher number of doses) and convenience of the therapeutic regimen [28].

The objective of the study reported here was to compare the annual duration of flea and tick treatment purchased by cat owners in the USA who were prescribed the long duration treatment of fluralaner with the duration purchased by cat owners prescribed monthly ectoparasiticides.

## Methods

### Study design

Client topical feline flea and tick treatment purchase transactions from veterinary clinics throughout the USA were evaluated retrospectively. Transaction records were obtained from a proprietary medical records data collection service (VetInformatics, Inc., Rolling Meadows, Ill, USA). Transactional record data did not contain information on the names or addresses of the cat, the owner or the veterinary clinic. Code numbers had been assigned which allowed serial transactions to be matched to individual feline patients without exposing the identity of the clinic or cat owner. These data were used to calculate the average annual number of doses purchased by cat-owners of each of three flea and tick medications. The number of doses purchased were then used to calculate the average duration of flea and tick protection for each medication based on the recommended protection duration for each treatment.

The study database (before exclusions) consisted of purchasing records from January 2017 through June 2019 from 671 veterinary practices representing approximately 41,630 cats. Feline flea and tick medications purchased by clients were determined for each individual feline patient for the 12-month period following the purchase index date, defined as the date of initial feline flea and tick medication purchase. The annual transactions were converted into a duration of coverage period based on the prescribing information for the respective flea and tick medication.

Flea and tick protection duration over the 12 months following the index date could not exceed the total doses purchased that could be reasonably used during this follow-up period. If owners obtained doses late in the 12-month follow-up period, then only the purchased doses that could be used by the end of the 12-month period were included in the coverage calculation. Therefore, the study only used purchased doses obtained in a 12-month period that could be used in 12 months for a specific cat. Owners who purchased multiple doses in a single transaction were assumed to have administered these doses on time and in a serial manner.

### Flea and tick treatments

The fipronil/S-methoprene/pyriproxyfen and Imidacloprid/ pyriproxyfen medications used for the study population (Table 1) require monthly re-dosing and one, fluralaner, is an extended duration ectoparasiticides with a retreatment interval of 12 weeks. In an online market research study of 250 USA vets, these products were some of the non-heartworm parasiticides for cats with the largest penetration into the USA veterinary market [29].

**Table 1:** Topical flea and tick medications used in U.S. feline dosage adherence study

Product characteristic	Bravecto^®^ Topical Solution	Frontline^®^ Gold	Advantage II^®^
Active ingredient	Fluralaner	Fipronil/S-methoprene/pyriproxyfen	Imidacloprid/pyriproxyfen
Redosing interval	12 weeks	1 month	1 month
Activity	Insecticidal for fleas, flea eggs and larvae Acaricidal for ticks	Insecticidal for fleas, flea eggs and larvae Acaricidal for ticks	Insecticidal for fleas, flea eggs, and larvae

### Inclusion and exclusion criteria

Product purchase data were screened to ensure that cats enrolled in the study received only one of the three flea and tick products for the 12-month study period, and that product purchases represented transactions for only one cat. Cats included in the study were ≥ 6 months-old on the index day. Transactions for medications that included endoparasite indications were excluded. Transactions for any species other than cats were excluded. Single transactions that demonstrated purchases in excess of 12 months of flea and tick protection were uncommon and were excluded as likely purchases for more than one cat. Product usage by individual owners was adjusted by removing any doses returned to the clinic for credit.

### Statistical evaluation

The mean age, age group, body weight and range were determined for cats enrolled in each product group. The flea and tick medication coverage obtained by cat owners was expressed as a population mean, standard deviation (SD), and percentage for each product and for products grouped by duration. Means were compared across groups using a t-test with significance set at *P* < 0.05.

## Results

### Test population demographics

Three comparator flea and tick medications were examined (Table 1). The geographical distribution of the veterinary clinics in this study was defined by the clinics that were included in the study database. Transaction records were obtained from veterinary clinics in the Southeast (50%), Midwest (21%), South Central (15%), West (8%) and Northeast (6%) USA. Demographic profiles for cats in each of the three treatment groups are similar (Table 2). Most cats (63.7%) were treated with fluralaner (*n* = 27,440) *versus* fipronil/s-methoprene/pyriproxyfen (*n* = 10,607; 24.5%), and imidacloprid /pyriproxyfen (*n* = 3583; 8.3%). Most cats in each treatment group, where a recorded age was available, were 1.1–8.0 years of age, although > 30% in each group were > 8 years of age and considered mature or senior cats. Most cats in each treatment group had a mean weight between 3.7–7.3 kg), where a recorded weight was available.

**Table 2:** Demographics of the cat population from U.S. veterinary practices participating in the topical ectoparasiticide coverage study

Demographic category	Bravecto^®^ Topical Solution	Frontline^®^ Gold	Advantage II^®^	All cats
No. of cats treated (%)	27,440 (65.9%)	10,607 (25.5%)	3583 (8.6%)	41,630 (100%)
Cat age (years)				
Mean ± SD	6.7 ± 4.7	6.9 ± 5.0	6.9 ± 5.1	6.7 ± 4.8
Median	5.5	5.8	5.6	5.6
No. of cats by age group				
6 months–1 year, *n* (%)	1132 (4.1)	537 (5.1)	211 (5.9)	1880 (4.5)
1.1–8.0 years, *n* (%)	16,493 (60.1)	6075 (57.3)	2041 (57.0)	24,609 (59.1)
8.1–12.0 years, *n* (%)	5185 (18.9)	1956 (18.4)	642 (17.9)	7783 (18.7)
> 12.0 years, *n* (%)	4373 (15.9)	1907 (18.0)	671 (18.7)	6951 (16.7)
Data missing	257 (0.9)	132 (1.2)	18 (0.5)	407 (1.0)
Cat weight (kg)				
Mean ± SD	4.5 ± 0.4	4.2 ± 0.4	4.0 ± 0.3	4.4 ± 0.4
Median	4.5	4.5	4.1	4.5
No. of cats by body weight group				
≤ 0.9 kg, *n* (%)	111 (0.4)	109 (1.0)	38 (1.1)	258 (0.6)
0.9–1.8 kg, *n* (%)	862 (3.1)	534 (5.0)	209 (5.8)	1605 (3.9)
1.9–3.6 kg, *n* (%)	7575 (27.6)	3128 (29.5)	1293 (36.1)	11,996 (28.8)
3.7–7.3 kg, *n* (%)	17,258 (62.9)	6218 (58.6)	1945 (54.3)	25,421 (61.1)
7.3–14.5 kg, *n* (%)	1144 (4.2)	297 (2.8)	64 (1.8)	1505 (3.6)
> 14.5 kg, *n* (%)	2 (0.01)	4 (0.4)	0 (0.0)	6 (0.01)
Data missing	488 (1.8)	317 (3.0)	34 (1.0)	839 (2.0)

### Annual purchasing history and flea and tick (F/T) treatment coverage

Annual client purchasing history and mean duration of coverage provided by the three F/T medications (Table 3) show that cat owners purchased more individual doses per year of each of the two ectoparasiticides which were dosed on a monthly basis compared to the number of fluralaner doses, which have an extended duration that is nearly 3-fold the length of the monthly dose. The extended duration of treatment coverage provided by fluralaner translated into significantly more months of annual flea and tick coverage (4.2 months) compared to the duration of coverage provided by fipronil/s-methoprene/pyriproxyfen (3.6 months; (*t*_(38,045)_ = 34.1481, *P* < 0.0001) and imidacloprid/pyriproxyfen (*t*_(31,021)_ = 50.3001, *P* < 0.0001). Annual purchases of fluralaner provided 17% and 50% longer duration of coverage *versus* fipronil/s-methoprene/pyriproxyfen, and imidacloprid/pyriproxyfen, respectively.

**Table 3:** Mean annual per-cat ectoparasiticide doses purchased by USA cat owners and mean months of flea and tick coverage provided^1^

Product prescribed	Bravecto^®^ Topical Solution (*n* = 27,440)	Frontline^®^ Gold (*n* = 10,607)	Advantage II^®^ (*n* = 3583)
Doses purchased (Mean ± SD)	1.52 ± 0.90^a^	3.58 ± 3.02^b^	2.81 ± 2.77^b^
Months F/T^a^ coverage per year (Mean ± SD)	4.20 ± 1.58^a^	3.58 ± 1.61^b^	2.81 ± 1.35^b^

^1^Numbers with different superscripts differ significantly. Mean months of coverage were significantly different between Bravecto Topical and Frontline Gold (*t*_(38,045)_ = 34.1481, *P* < 0.0001) as well as Bravecto Topical and Advantage II (*t*_(31,021)_ = 50.3001, *P* < 0.0001)

**Fig. 1:**
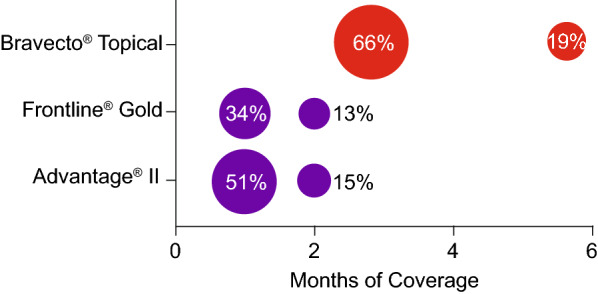
Cat owner purchases of one or two doses of flea and tick medication annually. Large circles indicate percentage purchasing a single dose. Small circles indicate percentage purchasing a second dose. The x-axis indicates the months of coverage provided by the doses purchased. The size of the circle is in proportion to the proportion of cat owners purchasing that product

Flea and tick treatment purchases were dominated by pet owners who purchased only one or two treatment doses per year, regardless of the medication duration of protection (Table 4, Fig. 1). Approximately 67% of cat owners who purchased fluralaner obtained one dose and approximately 18% obtained two doses. A large proportion of owners who bought monthly flea and tick treatments limited their annual purchases to either one or two doses as well (34% and 13%, respectively, for fipronil/s-methoprene/pyriproxyfen; 51% and 15%, respectively, for imidacloprid/pyriproxyfen). Two doses of fluralaner provide up to 5.6 months of treatment coverage, while two doses of a monthly flea and tick treatment provided up to 2 months of coverage.

**Table 4:** Months of feline flea and tick coverage acquired by USA cat owners based on annualized ectoparasiticide purchases

Months of coverage	Bravecto^®^ Topical Solution-treated cats % (*n*)	Frontline^®^ Gold-treated cats % (*n*)	Advantage^®^ II-treated cats % (*n*)
1.0–1.9		33.6 (3578)	51.2 (1836)
2.0–2.9	67.0 (18,394)^a^	13.1 (1397)	14.7 (527)
3.0–3.9	0.7 (180)	17.6 (1871)	6.8 (244)
4.0–4.9	0.6 (159)	9.3 (993)	8.2 (292)
5.0–5.9	18.1 (4968)	2.1 (218)	1.8 (66)
6.0–6.9	0.8 (211)	9.8 (1045)	8.4 (302)
7.0–7.9	1.9 (514)	1.5 (157)	1.2 (43)
8.0–8.9	4.7 (1276)	3.8 (409)	2.3 (81)
9.0–9.9	0.7 (179)	2.5 (265)	0.5 (19)
10.0–10.9	2.2 (595)	0.7 (76)	0.5 (18)
11.0–11.9	3.5 (964)	0.6 (58)	0.5 (19)
12.0		5.1 (540)	3.8 (136)

^a^One dose of Bravecto Topical Solution (fluralaner) provides 12 weeks, or 2.79 months, of coverage

The distribution of the months of treatment coverage based on annual ectoparasiticide dose purchases is shown (Table 4). A small proportion (8–10%) of cat owners who purchased monthly products obtained 6 doses (6 months) of flea and tick treatment coverage. While veterinarians often recommend a full year of flea protection, a full year of treatment coverage was obtained by an even smaller proportion of cat owners across all brands.

## Discussion

The three ectoparasiticides selected for comparison in this study (Table 1) made up the largest proportion of the feline ectoparasiticides provided to cat owners (excluding combinations with endoparasitic activity) as recorded by the veterinary practice transaction database in the USA for the study period. All three medications are labelled to treat fleas but only fluralaner and fipronil/s-methoprene/pyriproxyfen are also acaricidal.

Medication purchasing data are a reliable surrogate for medication adherence [30], as long as the purchased medication is administered as recommended. Purchased medication may not be administered as recommended, which means adherence measured from medication purchases would then overestimate true adherence by an unknown amount. A medication administration adherence failure could occur each time the dose needs to be administered. Therefore, more doses to administer leads to a greater probability of failure. This helps to explain why the need to administer fewer doses of a longer acting medication consistently leads to better adherence with prescriber recommendations.

Demographic data (Table 2) for participating cats confirm that the study population was similar in age and weight across treatment groups. Cats had a mean age that varied by ≤ 3 months (6.7–6.9 years) and a mean weight that varied by ≤ 0.5 kg (4.0–4.5 kg) across all treatment groups.

Prior studies comparing dog owner flea and tick medication purchases have shown that owners obtain more months of coverage when they buy an extended duration product [26, 31]. This study confirms that cat owners similarly will obtain more annual months of flea and tick coverage compared to owners that obtain products with a monthly retreatment interval. Cat owners purchased up to 50% longer duration of flea and tick protection per year when they purchased fluralaner, compared with owners who purchased monthly flea and tick medications.

Owner purchases of any medication can be compared with veterinary recommendations to get an assessment of medication adherence. Earlier studies found that veterinarians in the USA recommend 12 months of protection each year against fleas and ticks [8, 30]. Cat owners increased their adherence to this recommendation through use of a product with a longer duration of action. This is also consistent with prior data showing that treatment adherence is inversely related to dosing frequency, a critical convenience factor [28, 32]. Even with the benefit of extended duration flea and tick medication, cat owners in the USA fall well short of full adherence with veterinary recommendations for flea and tick protection duration.

Cats are the most popular pet in the USA [33], but they receive much less primary veterinary medical care compared to dogs, and 40% of pet cats in one study had not seen a veterinarian during the preceding year compared to 15% of dogs [34]. Pet owners surveyed in 2006 took their dogs to the veterinarian an average of 2.3 times per year compared to 1.1 times/year for cats [35]. This healthcare underutilization for cats could make poor adherence to treatment recommendations more likely. Therefore, any delivery system that increases owner adherence is likely to improve the preventive health care for their cats.

Year-round flea and tick treatment coverage for cats directly protects the cat, eradicates flea populations in the home environment [13], and helps to reduce the risk of vector transmitted disease [1]. However, as seen in this study, cat owners typically acquired only one or two treatment doses, regardless of the duration of the flea and tick medication purchased and few owners obtained full-year flea and tick coverage (Table 4). Therefore, veterinary clinicians and practice staff may want to pay increased attention to owner education and use of repurchase reminders as additional components that contribute to increased owner adherence.

These results for cat owners are consistent with previous results in a large group of USA dog owners [36]. Dog owners purchased fewer doses and obtained longer flea and tick treatment coverage per year from fluralaner compared with monthly medications. Similar results were reported in a large-scale European study comparing owner-purchased canine flea and tick medications, comparing oral fluralaner and several monthly oral or topical products [31]. Dog owners in Spain purchased fewer fluralaner doses per year compared to monthly oral or topical medications but obtained significantly more flea and tick coverage. Collectively, these studies confirm that pet owners obtain a relatively longer duration of flea and tick control by use of longer duration flea and tick treatments.

## Conclusions

Cat owners who obtained an ectoparasiticide with a 12-week redosing interval protected their cats for a longer period during the year than owners who purchased a monthly treatment. Veterinarians and hospital staff should help cat owners increase the duration of flea and tick protection they obtain to achieve veterinary recommendations and to optimize protection against ectoparasites [26].

## Data Availability

The datasets were obtained from a public source (VetInformatics, Inc., Rolling Meadows, Ill, USA). The analysis was generated during the current study and is not publicly available because this is the proprietary property of Merck & Co., Inc., Kenilworth, NJ, USA, Inc.

## Abbreviations

CAPC: Companion Animal Parasite Council
F/T: Flea and tick
